# Diagnostic accuracy of endoscopic ultrasound-guided fine-needle aspiration: A single-center analysis

**DOI:** 10.7150/ijms.48882

**Published:** 2020-10-16

**Authors:** Songming Ding, Aili Lu, Xinhua Chen, Bingqian Xu, Ning Wu, Muhammad Ibrahim Alhadi Edoo, Shusen Zheng, Qiyong Li

**Affiliations:** 1 Shulan (Hangzhou) Hospital Affiliated to Zhejiang Shuren University, Shulan International Medical College, #848 Dongxin Road, Hangzhou, Zhejiang, P.R. China.; 2 Division of oncology department, First Affiliated Hospital, Zhejiang University School of Medicine, Hangzhou, Zhejiang, P.R. China.; 3 Division of Hepatobiliary and Pancreatic Surgery, First Affiliated Hospital, ZhejiangUniversity School of Medicine, Hangzhou, Zhejiang, P.R. China.

**Keywords:** Endoscopic ultrasound, fine needle aspiration biopsy, cytology, pancreas

## Abstract

**Background:** Endoscopic ultrasound-guided fine-needle aspiration biopsy (EUS-FNAB) has become an important modality for identification of intra-abdominal masses. This study analyzed the accuracy of EUS-FNAB in a single medical center and explored factors related to positive diagnosis.

**Materials and methods:** In total, 77 patients with EUS-FNAB were retrospectively reviewed from July 2016 to February 2020. “Atypical (tends to be neoplasm/malignancy),” “suspicious (first consider neoplasm/malignancy),” and “malignant” were defined as positive cytology. The final diagnoses were based on histopathologic examination. The positive rate of EUS-FNAB for the diagnosis of neoplasm and its associations with age, sex, target puncture mass size, liver function, tumor markers, albumin, hypertension, and diabetes were examined.

**Results:** Accuracy, sensitivity, specificity, positive predictive value, and negative predictive value of EUS-FNAB cytologic diagnoses in all patients were 77.9% (60/77), 76.1% (54/71), 100%, 100%, and 26.1% (6/23), respectively. Accuracy, sensitivity, specificity, positive predictive value, and negative predictive value of EUS-FNAB cytologic diagnoses in the pancreas were 80.0% (48/60), 79.3% (46/58), 100%, 100%, and 14.3% (2/14), respectively. The results of EUS-FNAB in pancreatic masses showed that the level of CA19-9 was higher in the true positive group than in the false-negative group (*p*<0.05). There were no factors associated with the true positive cytologic diagnoses (*p*>0.05).

**Conclusions:** Our single-medical center study showed that EUS-FNAB is an accurate diagnostic procedure for the evaluation of intra-abdominal masses. Further follow-up is required to explore factors associated with the true positive cytology.

## Introduction

Endoscopic ultrasound-guided fine-needle aspiration biopsy (EUS-FNAB) was originally introduced in the early 1990s 1, 2. It was first clinically applied in patients with stomach subepithelial lesions, then in patients with pancreatic disease 3. It is currently used worldwide. EUS-FNAB is considered a safe medical tool with morbidity and mortality rates <1%. Notably, its sensitivity is 60%-95% and specificity is 71%-100% (based on previous reports, the overall diagnostic accuracy ranges from 60% to 90%) 4-6. Accurate diagnoses obtained according to the cytopathological results of EUS-FNAB include pancreatic duct adenocarcinoma 7, 8, pancreatic neuroendocrine tumors 9-11, intra-abdominal lymphoma 12-14, gastrointestinal stromal tumors 15-17, peripancreatic tuberculous lymphadenitis 18, 19, autoimmune pancreatitis 20, and chronic pancreatitis.

The diagnostic rate of EUS-FNAB is reportedly dependent on numerous factors such as mass characteristics (location, size, and echogenicity), needle type, number of passes, stylet and suction, rapid on-site evaluation (ROSE) by an experienced cytopathologist, and endosonographer experience and skill 21-25. However, clinicians continue to encounter false-negative or even false-positive results 26-29. Ongoing studies focus on the use of new puncture needles to obtain adequate samples and preserve tissue architecture 30, 31. Pathologists promote the ROSE of samples to improve the diagnostic yield 32.

Our hospital is a tertiary referral center. The complexity and diversification of diseases often requires multi-disciplinary cooperation. The combinations of EUS-guided fine-needle aspiration, body surface ultrasound-guided fine-needle aspiration, and laparoscopic biopsy are highly respected by our center. We have not yet applied the new needle and rapid cytopathological evaluation.

The purpose of this study was to analyze the accuracy of EUS-FNAB at our center and explore factors related to positive diagnosis. The positive rate of EUS-FNAB for the diagnosis of neoplasm and its relationships with age, sex, target puncture mass size, liver function (transaminase, alkaline phosphatase, γ-glutamyl transferase, serum total bilirubin, and direct bilirubin), tumor markers (CA19-9, CEA, AFP, CA125, and ferritin), albumin, hypertension, and diabetes were examined.

## Patients and methods

This single-center retrospective study was conducted at Shulan (Hangzhou) Hospital, Affiliated with Shulan International Medical College, Zhejiang Shuren University, Hangzhou, P.R. China, from July 2016 to February 2020. The study protocol was approved by the ethics committee of Shulan (Hangzhou) Hospital (number: 2020014). In total, 77 patients were enrolled in the study and their medical records were reviewed. All patients withdrew anticoagulants for at least 1 week and fasted for more than 4-6 h before the procedure.

Patients were placed in the left lateral decubitus position with tooth protection and were sedated with intravenous anesthesia and dexmedetomidine administration, with opioids for analgesia. Oxygen was supplied via the nasal cannula; no patients required endotracheal intubation. Vital signs were recorded continuously. All procedures were performed using a linear array echoendoscope (GF UCT260; Olympus Medical Systems, Tokyo, Japan) connected to an ultrasound scanning system (EU-ME2 PREMIER PLUS; Olympus Medical Systems, Tokyo, Japan) by three experienced endosonographers, who were assisted by an endoscopic nurse. Following careful scope manipulations and identification of the target puncture mass, the location, size, shape, and echogenicity were carefully assessed and recorded. Color and Doppler sonography was performed to avoid vascular structures and to select a vessel-free needle track using a standard 19G (n = 2), 22G (n = 73), or 25G (n = 2) aspiration needle (Boston Scientific Expect^TM^, USA or Wilson-Cook Medical ECHO, USA). Upon visualization of the tip of the catheter, the needle was advanced from the catheter sheath through the wall of the duodenum or stomach. After the needle had successfully entered the target puncture mass, its stylet was withdrawn and suction was applied using a 10-ml syringe. Finally, the needle was removed from the mass after suction had been released 33, 34. The number of passes depended on the endosonographer's estimation of the yielded material and ease of the operation (for this study, at least two passes were performed).

Alcohol-fixed smears and liquid-based slides were prepared routinely for cytologic pathology (Papanicolaou or hematoxylin-eosin staining). Histologic examinations were also performed when additional material was available (n = 40; 15 also had postoperative pathology or other biopsy pathology, and the results were consistent); materials were fixed in 10% buffered formalin liquid, Papanicolaou, or hematoxylin-eosin staining solution. Immunohistochemical results were available for a few patients; “atypical (tends to be malignancy or tumorigenicity),” “suspicious (first consider malignancy or tumorigenicity),” and “malignant” were defined as positive cytologic diagnoses. The final diagnosis was defined based on the following criteria: (1) Neoplastic lesions, histopathologic diagnosis obtained based on surgery resected samples (n=16) or biopsy (n=34), and clinical diagnosis as neoplasm based on clinical follow-up of symptoms, imaging performance, and tumor markers (n=21). Among the neoplastic lesions, seven did not have positive cytology (one pancreatic neuroendocrine tumor, one intraductal papillary mucinous tumor of pancreas, one ampullary carcinoma with liver metastasis, three pancreatic cancer with surrounding tissue invasion, and one cholangiocarcinoma hilar recurrence). (2) Benign lesions (n=6), benign cytopathologic/histopathologic findings and clinical follow-up with no evidence of malignant progression or metastasis; antituberculotic treatment was effective. Our hospital is a referral center for patients with complicated disease, and all patients have a previous history of hospital admission; therefore, the clinical follow-up interval was at least 3 months.

Independent Student's *t*-tests or the Mann-Whitney U test were used to compare differences continuous variables between the two groups. Chi square test was used to compare the differences of categorical variables between the two groups. Risk factors were assessed using Binary logistic regression. The level of statistical significance for all tests was defined as *p*<0.05.

## Results

In this study, 77 patients with EUS-FNAB were included, among which 60 FNABs were taken from the pancreas (37 head/uncinate process vs 23 body/tail) and 17 were taken from extrapancreatic intra-abdominal sites. The basic characteristics are shown in **Table 1.** The mean ages of all patients, patients in the pancreas group, and patients in the extrapancreatic group were 60.95 years, 61.10 years, and 60.41 years, respectively. The ratios of men/women among all patients, patients in the pancreas group, and patients in the extrapancreatic group were 3.1, 5.0, and 0.9, respectively. No obvious adverse events associated with EUS-FNAB, such as gastrointestinal tract perforation or intra-abdominal bleeding, were reported. A total of 71 neoplastic lesions and six benign lesions were identified, as shown in **Table 2.** Pancreatic adenocarcinoma was the most common lesion (n=46), followed by pancreatic neuroendocrine tumors (n=5). False-negative findings are also listed in **Table 2.** Six benign masses were interpreted as one mass-forming pancreatitis, one chronic pancreatitis with pseudocyst, one reactive lymph node hyperplasia after drug-induced liver transplantation, one intraabdominal fibrocalcified nodule with reactive lymph node hyperplasia, and two with intraperitoneal tuberculosis. Accuracy, sensitivity, specificity, positive predictive value (PPV), and negative predictive value (NPV) findings of EUS-FNAB cytologic diagnoses in all patients were 77.9% (60/77), 76.1% (54/71), 100%, 100%, and 26.1% (6/23), respectively. Accuracy, sensitivity, specificity, PPV, and NPV findings of EUS-FNAB cytologic diagnoses in the pancreas were 80.0% (48/60), 79.3% (46/58), 100%, 100%, and 14.3% (2/14), respectively. Accuracy, sensitivity, specificity, PPV, and NPV findings of EUS-FNAB cytologic diagnoses in extrapancreatic intra-abdominal sites were 70.6% (12/17), 61.5% (8/13), 100%, 100%, and 44.4% (4/9), respectively, as shown in **Table 3.**

**Figure 1:**
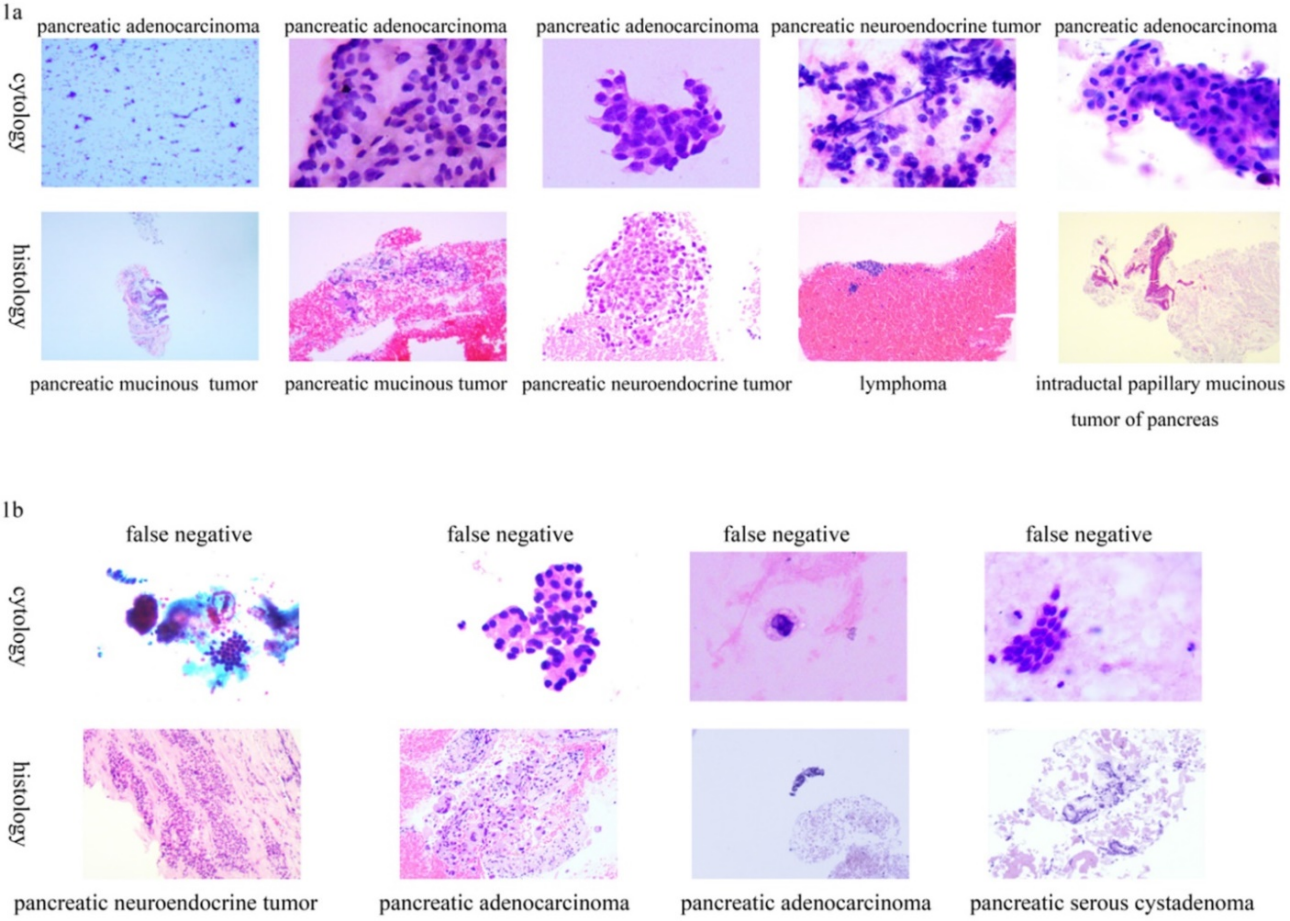
Inconsistent cytology results with histological diagnosis using endoscopic ultrasound-guided fine-needle aspiration biopsy. 1a): Cytology true positive; 1b): Cytology false negative.

Five patients had inconsistent cytological and histological diagnoses, as shown in **Table 4.** The cytological results are shown in **Figure 1a.** Four patients had no positive cytological results, but exhibited positive histological results of EUS-FNAB, as shown in **Table 4.** Their pathological results are shown in **Figure 1b.**

**Figure 2:**
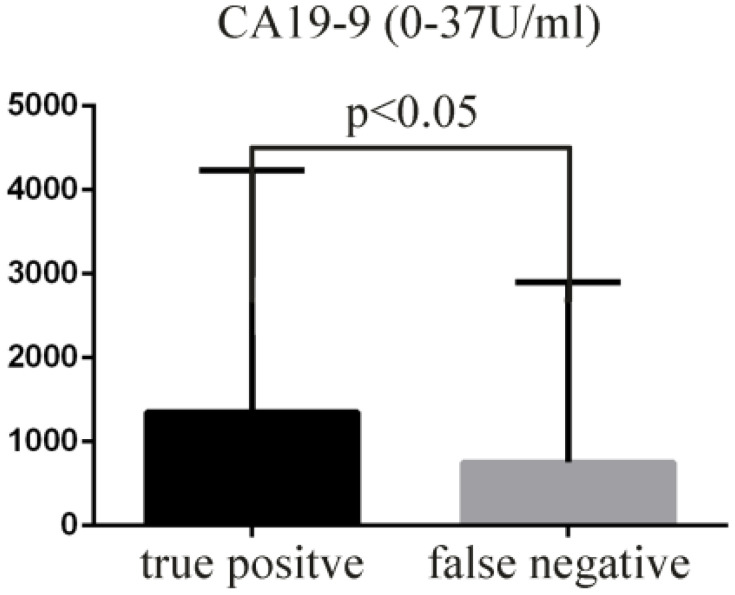
Comparison of the level of tumor marker CA19-9.

The true positive rate of EUS-FNAB in the diagnosis of neoplasm and its relationships with age, sex, target puncture mass size, liver function (transaminase, alkaline phosphatase, γ-glutamyl transferase, serum total bilirubin, direct bilirubin), tumor markers (CA19-9, CEA, AFP, CA125, ferritin), albumin, hypertension, and diabetes were examined. The above-mentioned factors were not correlated with the true positive cytologic diagnoses (*p*>0.05). Only the level of CA19-9 was higher in the true positive group (mean ± SD, 1350.85 ± 2878.46) than in the false-negative group (mean ± SD, 750.06 ± 2152.99) (**Figure 2**, *p*<0.05).

## Discussion

EUS has two scopes: radial (for evaluation of the positional relationship with surrounding organs) and longitudinal (for evaluation of the relationship between the target lesion and nearby blood vessels). To detect pancreatobiliary tumors, gastrointestinal stromal tumors, and other tumors from the upper digestive tract, EUS has shown superiority to CT scans, surface ultrasound, and endoscopic retrograde cholangiopancreatography 35-37. However, EUS cannot provide cytopathologic or histopathological diagnoses; thus, there is a specific rate of misdiagnosis. In particular, EUS cannot distinguish benign lymph node hyperplasia from malignant lymph node metastasis. Based on this limitation, FNAB under EUS guidance was designed. After nearly 30 years of development, EUS-FNAB is now able to diagnose pancreatic duct adenocarcinoma 7, 8, pancreatic neuroendocrine tumors 9-11, 38, pancreatic cystic lesions 39, intra-abdominal lymphomas 12-14, gastrointestinal stromal tumors 15-17, peripancreatic tuberculous lymphadenitis 18, 19, and pancreatitis 20.

Notably, EUS-FNAB includes EUS-fine-needle aspiration and EUS-fine-needle biopsy. EUS-fine-needle aspiration can be used to obtain cellular samples for cytological diagnosis, but does not typically retain the stroma or associated architecture, which complicates the acquisition of a definite diagnosis of malignancy; EUS-fine-needle biopsy typically improves the procurement of samples with preserved tissue architecture and has become an indispensable tool in establishment of a diagnosis of malignancy 25, 40. The diagnostic accuracy of EUS-fine-needle biopsy is reportedly independent of the number of needle passes or the absence of ROSE 40, 41. Furthermore, EUS-fine-needle biopsy is known to outperform fine-needle aspiration in all diagnostic outcomes evaluated in subepithelial lesions 42. However, a network meta-analysis showed that no specific EUS-guided tissue sampling technique was superior with regard to diagnostic accuracy, sample adequacy, or histologic procurement rate for solid pancreatic masses 43. Nonetheless, sufficient tissue acquisition is important for EUS-FNAB.

In the present study, we reported our experience involving 77 patients who underwent EUS-FNABs in our hospital. Fifty patients had a subsequent histopathologic assessment and 21 patients had a clinical follow-up for neoplastic lesions. The clinical follow-up was reliable and 14 patients showed positive cytology results (10 were pancreatic adenocarcinoma with surrounding invasion or extensive lymph node metastasis, one was invasive gastrointestinal stromal tumor, one was esophageal cancer with extensive intraperitoneal metastasis, one was pancreatic neuroendocrine tumor, and one was intra-abdominal lymphoma). EUS-FNAB cytology findings in patients with intra-abdominal lymphoma suggested lymphocyte-like cells; morphologically naive lymphoma could not be excluded (Supplementary Material, Figure S1a). Enhanced MRI revealed multiple nodules and masses in the hilar area, hepatogastric space, and around the head and neck of the pancreas and retroperitoneum. Therefore, diagnosis of lymphoma is possible. EUS-FNAB cytology can also be used for pancreatic neuroendocrine tumors. EUS-FNAB cytology suggested that for scattered cells with a uniform size, cytosolic granular, nuclear bias, and focal rose pattern structure, a diagnosis of pancreatic neuroendocrine tumor should be considered (Supplementary Material, Figure S1b). Seven of the final diagnoses based on clinical follow-up showed no positive cytology results; one of these was pancreatic neuroendocrine tumor (typical imaging performance), one was an intraductal papillary mucinous tumor of the pancreas (typical imaging performance), one was an ampullary carcinoma with liver metastasis, three were pancreatic adenocarcinoma with surrounding tissues invasion, and one was a hilar recurrence of cholangiocarcinoma. Therefore, there were 71 neoplastic lesions and six benign lesions. There were no false-positive findings.

In our study, the accuracy, sensitivity, specificity, PPV, and NPV findings of EUS-FNAB cytologic diagnoses in all patients were 77.9% (60/77), 76.1% (54/71), 100%, 100%, and 26.1% (6/23), respectively. Accuracy, sensitivity, specificity, PPV, and NPV findings of EUS-FNAB cytologic diagnoses in the pancreas were 80.0% (48/60), 79.3% (46/58), 100%, 100%, and 14.3% (2/14), respectively. Accuracy, sensitivity, specificity, PPV, and NPV findings of EUS-FNAB cytologic diagnoses in extrapancreatic intra-abdominal sites were 70.6% (12/17), 61.5% (8/13), 100%, 100%, and 44.4% (4/9), respectively. Although the accuracy, sensitivity, specificity, PPV, and NPV of EUS-FNAB cytologic diagnoses were slightly lower in the extrapancreatic group, these results were not significantly different from those of previous studies reporting the sensitivity (60%-95%), specificity (71%-100%), and accuracy (60%-90%) of this procedure 4-6. In the extrapancreatic group, 13 were neoplasms (two gastrointestinal stromal tumors, one ampullary carcinoma with liver metastasis, one inflammatory myofibroblastic tumor based on post-operation pathology, two advanced gallbladder cancer, one extensive lymph node metastasis of esophageal squamous cell carcinoma, one extensive lymph node metastasis of colon cancer, one intra-abdominal lymphoma, one cholangiocarcinoma hilar recurrence, one extensive lymph node metastasis of penile cancer, and two extensive lymph node metastases of pancreatic cancer). There were five false-negative findings (29.4%): one was an inflammatory myofibroblastic tumor, one was a gastrointestinal stromal tumor, one was an ampullary carcinoma, one was a pancreatic cancer, and one was a hilar recurrence of cholangiocarcinoma. Three samples were obtained from lymph nodes, one was obtained from the hilum of the liver, and one was obtained from the space between the spleen and stomach. The small study population was the main reason for low accuracy and sensitivity in the extrapancreatic group. In the pancreas group, there were 12 false-negative findings (20%): one was an intraductal papillary mucinous tumor of the pancreas based on clinical follow-up, one was a pancreatic neuroendocrine tumor with positive histopathology by EUS-FNAB, one was a pancreatic neuroendocrine tumor based on clinical follow-up, one was a pancreatic serous cystadenoma with positive histopathology by EUS-FNAB, two were pancreatic adenocarcinoma with positive histopathology by EUS-FNAB, three were pancreatic adenocarcinoma based on clinical follow-up, one was a pancreatic adenocarcinoma based on laparoscopic biopsy pathology, one was a pancreatic mucinous tumor based on post-operation pathology, and one was a diffuse large B-cell lymphoma by liver biopsy. The primary cause of false negatives in the pancreatic group was acquisition of insufficient sample. In addition, accurate and timely performance of the cell smear after sample acquisition is an important concern, because there are inconsistencies between cytological and histological results.

At this time, the use of new puncture needles that can obtain adequate samples and the promotion of ROSE are important for improvement of diagnostic yield 25, 30-32. There is no availability of ROSE or the new type of puncture needle at our center. We explored the positive rate of EUS-FNAB for the diagnosis of neoplasm and its relationships with age, sex, target puncture mass size, liver function (transaminase, alkaline phosphatase, γ-glutamyl transferase, serum total bilirubin, direct bilirubin), tumor markers (CA19-9, CEA, AFP, CA125, ferritin), albumin, hypertension, and diabetes. The positive rate of liver function damage (especially obstructive jaundice) in patients with pancreatic cancer might have been high due to rapid progression of pancreatic cancer. The positive rate of hypoproteinemia in patients with pancreatic cancer might have been low due to edema, which is not conducive to acquisition of samples. Patients with metabolic diseases, such as hypertension and diabetes, may exhibit insufficient sample collection, which may have affected the positive cytology rate. However, this study did not show that the above-mentioned factors were correlated with true positive cytologic diagnoses (*p*>0.05). The level of CA19-9 was higher in the true positive group than in the false negative group (**Figure 2**, *p*<0.05) because CA19-9 is a marker of pancreatic ducal adenocarcinoma; however, it is not necessarily expressed in pancreatic neuroendocrine tumors, lymphoma, and some pancreatic cystic tumors.

## Conclusion

Our single-medical center data showed that EUS-FNAB is an accurate diagnostic procedure for the evaluation of deep-site intra-abdominal masses, especially for pancreatic masses. Achievement of a sufficient sample size is important for this technology. Further studies should include more patients for the investigation of factors related to positive cytology findings.

## Supplementary Material

Supplementary figure S1

## Figures and Tables

**Table 1:** The characteristics of the 77 patients included in the study

Characteristics	Total patients (n=77)	Pancreas (n=60)	Extra-pancreas (n=17)
Age (mean ± SD), years	60.95±12.82	61.10±10.65	60.41±19.03
Sex (male)	58	50	8
Mass size (cm), mean ± SD	3.64±1.63	3.69±1.57	3.45±1.88
Alanine aminotransferase (5-40 U/L)	57.64±91.46	59.75±96.78	50.18±71.57
glutamic oxaloacetic transaminase (8-40 U/L)	47.90±64.98	47.00±61.13	51.06±79.14
alkaline phosphatase (40-150 U/L)	162.89±186.41	146.32±144.76	220.41±287.06
γ-Glutamyl transferase (11-50 U/L)	281.79±958.74	173.75±293.66	656.76±1950.29
serum total bilirubin (0-21 umol/L)	43.52±69.71	42.02±63.16	48.82±91.31
serum direce bilirubin (0-5 umol/L)	30.81±58.08	28.78±51.40	37.94±78.81
Albumin (35-55 g/L)	39.49±4.58	40.03±4.49	37.59±4.51
CA19-9 (0-37 U/ml)	1396.54±3031.82	1183.38±2696.73	2234.95±4105.66
CEA (0-5 ng/ml)	11.10±27.93	8.56±14.89	20.92±54.58
AFP (0-20 ng/ml)	79.13±654.08	98.98±733.79	2.38±0.92
CA125 (0-35 U/ml)	88.29±231.73	51.87±104.33	239.18±463.82
ferritin (7-323 ng/ml)	559.3±587.54	554.91±595.24	577.81±575.02
hypertension and/or diabetes (yes)	31	25	6

**Table 2:** Intra-abdominal lesions nature

Neoplastic lessions	n	Final gold diagnoses criteria 1	Final gold diagnoses criteria 2	Final gold diagnoses criteria 3	Final gold diagnoses criteria 4	False negative lesions
Pancreatic adenocarcinoma	46	27	11	5	13	7
Pancreatic neuroendocrine tumor	5	3	2		2	2
Pancreatic serous cystadenoma	1	1	1			1
Pancreatic mucinous (cystic ) tumor	3	2	1			1
Solid-pseudopapillary tumor of pancreas	1	1				
Intraductal papillary mucinous tumor of pancreas	2	1			1	1
Cholangiocarcinoma hilar recurrence	1				1	1
Gastrointestinal stromal tumor	2	1		1	1	1
Lymphoma	3	1		2	1	1
Gallbladder cancer	2	2				
Colon cancer	1			1		
Ampullary carcinoma	1				1	1
Esophageal cancer	1				1	
Penile cancer	1	1				
Inflammatory myofibroblastic tumor	1		1			1
Benign lesions	6					
Pancreatitis	2				2	
Intraperitoneal tuberculosis	2	1			1	
Reactive lymph node hyperplasia after drug-induced liver transplantation	1				1	
Intraabdominal fibrocalcified nodule with reactive lymph node hyperplasia	1		1			
Final gold diagnoses criteria 1 based on EUS-FNAB histopathology						
Final gold diagnoses criteria 2 based on postoperative pathology						
Final gold diagnoses criteria 3 based on histopathology of liver biopsy or other biopsies						
Final gold diagnoses criteria 4 based on clinical follow-up						

**Table 3:** The accuracy, sensitivity, specificity, PPV and NPV of EUS-FNAB

	All intra-abdominal masses	Pancreas	Extra-pancreas
True positive	54	46	8
True negative	6	2	4
False negative	17	12	5
Accuracy (%)	77.9	80	70.6
Sensitivity (%)	76.1	79.3	61.5
Specificity (%)	100	100	100
PPV (%)	100	100	100
NPV (%)	26.1	14.3	44.4

**Table 4:** Inconsistent cytological with histological diagnosis

Cytological diagnosis	Histological diagnosis	Immunohistochemical results	n
Pancreatic adenocarcinoma	Pancreatic mucinous tumor	CK19(+), CK7(+), CA199(+), Mucin5AC(+), CEA(+), Ki-67(10%+), Villin(+)	2
Pancreatic adenocarcinoma	Intraductal papillary mucinous tumor of pancreas	CK7(+), CA199(+), Mucin5AC(+), Ki-67(40%+), CDX2(+), EMA(+), CAM5.2(+)	1
Pancreatic neuroendocrine tumor	Lymphoma	CgA(-), Syn(-), CD56(-), Ki-67(90%+), CK(P)(-), CK19(-), CK7(-), P53(-), CD20(+), CD79a(+), BCL-6 (+60%), BCL-2 (+50%), MUM1 (+20%), C-myc (+80%)	1
Pancreatic adenocarcinoma	Pancreatic neuroendocrine tumor	Syn(+), CgA(+), CK7(+), CK19(+)	1
False negative	Pancreatic neuroendocrine tumor	Syn(+), CgA(+-), CA199(-)	1
False negative	Pancreatic adenocarcinoma	CK7(+), CK19(+), CA199(+)	2
False negative	Pancreatic serous cystadenoma	CK19(+), CEA(+), MUC5(AC)(+), EMA(+), HNF1B(+) ,PAX-8(-), CK8/18(+), Inhibin-α(+)	1
