# Supplementary material for: Diagnostic accuracy of endoscopic ultrasound-guided fine-needle aspiration: A single-center analysis
Source: Int J Med Sci. 2020 Oct 16;17(17):2861–8. doi: 10.7150/ijms.48882 (PMC7645325; doi:10.7150/ijms.48882)

Supplement Figure S1

Figure. S1 Representative cases of specimens obtained by using endoscopic ultrasound-guided fine-needle aspiration biopsy. S1a): Lymphoma; S1b) Pancreatic neuroendocrine tumor.

Figure. S1

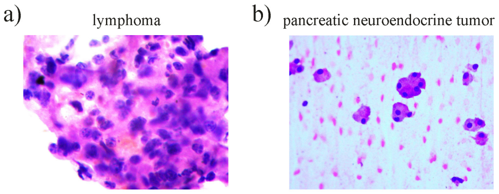

Supplement: Supplementary file 1 — Supplementary figure S1 [file ijmsv17p2861s1.pdf]
